# The Relationship Between Attention Deficit Hyperactivity Disorder and Major Depressive Disorder in Young Adults: Mediating Role of Mood and Anxiety Disorder Symptoms

**DOI:** 10.1192/j.eurpsy.2025.790

**Published:** 2025-08-26

**Authors:** D. U. Erarslan, H. Yapıcı Eser, S. Hun Şenol, A. Ercan Doğan

**Affiliations:** 1 Psychiatry, Koc University Faculty of Medicine, Istanbul, Türkiye

## Abstract

**Introduction:**

Attention Deficit Hyperactivity Disorder (ADHD) is a lifetime co-diagnosis of one or more psychiatric disorders in 70-80% of cases. In adults, ADHD is associated with anxiety, MDD, bipolar disorder and substance use disorder. (Faraone, S.V. *et al., Nature Reviews Disease Primers* (2024)). The aim of this study was to examine the relationship between ADHD and MDD symptoms in young adults and the mediation of mood and anxiety disorder symptoms on this relationship.

**Objectives:**

In this study, we examined the explainability of the relationship between ADHD and MDD symptoms in young adults through mood and generalized anxiety disorder scales. The aim was to reveal the relationships between different psychiatric dimensions in young adults presenting with these symptoms and to present the necessary treatment approaches.

**Methods:**

Between September 2019 and September 2022, 935 students who applied to Koç University Psychological Counseling and Psychotherapy Center were examined. Sociodemographic information and data were collected using the Adult ADHD Self-Report Scale (ASRS-v1.1), Patient Health Questionnaire-9 (PHQ-9), Generalized Anxiety Disorder-7 (GAD-7) and Mood Disorder Questionnaire (MDQ). Data were evaluated with descriptive statistics and Pearson correlation analysis. The effect of ASRS on PHQ-9 and the mediating effect of GAD-7 and DSQ scores were analyzed with Process macro model 4. The study was approved by Koç University Ethics Committee (Protocol No: 2024.291.IRB2.124).

**Results:**

82.8% of the students were classified as ADHD positive according to the ASRS 24 cut-off score. According to PHQ-9, the rate of depression was 78.1%. The rate of ADHD and depression positives was 70% according to ASRS24, 57.6% according to ASRS30 and 22.8% according to ASRS44. In the mediation analysis in which ASRS, PHQ9, GAD7 and MDQ scales were evaluated, the model explained 52% of the variance (p<0.00001). The direct effect of ASRS on PHQ9 (β: 0.35; p<0.00001; 95% CI: 0.29-0.39) was higher than the indirect effects via GAD7 (β: 0.21; 95% CI: 0.175-0.245) and DBQ (β: 0.025; 95% CI: 0.0027-0.048); the effect of MDQ was minimal.

**Conclusions:**

The study shows that ADHD symptoms are frequently associated with depression and that anxiety and mood disorder symptoms may strengthen this association. The possible bidirectional relationship between disorders should be considered in young adulthood assessment and should be followed up with longitudinal studies. The strengths of the study are its large sample size and focus on a significant age range; limitations are its cross-sectional nature and reliance on self-report scales.

**Disclosure of Interest:**

None Declared

